# Ultrasound-assisted uniform deposition of Au nanoparticles on biomass derived activated carbon for high performance supercapacitors^☆^

**DOI:** 10.1016/j.ultsonch.2026.108104

**Published:** 2026-10-09

**Authors:** Tippapon Tocuweang, Darunee Aussawasathien, Hirokazu Okawa, Nakorn Worasuwannarak

**Affiliations:** a The Joint Graduate School of Energy and Environment, Center of Excellence on Energy Technology and Environment, King Mongkut’s University of Technology Thonburi, Bangmod, Tungkru, Bangkok 10140, Thailand; b Advanced Polymer Technology Research Group, National Metal and Materials Technology Center, 114 Thailand Science Park, Phahonyothin Rd., Khlong Nueng, Khlong Luang, Pathum Thani 12120, Thailand; c Graduate School of Engineering Science, Akita University, 1-1 Tegatagakuen-machi, Akita 010-8502, Japan

**Keywords:** Soluble, Activated carbon, Supercapacitor, Ultrasound irradiation, Gold nanoparticle

## Abstract

Biomass-derived activated carbon is a promising sustainable electrode material for supercapacitors; however, improving its electrochemical performance requires effective surface modification while preserving its porous structure. Gold nanoparticles (AuNPs) are attractive modifiers, but their effectiveness strongly depends on particle size and dispersion. In this study, high-frequency ultrasonic irradiation (200 kHz) generates acoustic cavitation to promote AuNP deposition on rice-straw-soluble-derived activated carbon, and its effect is compared with conventional magnetic stirring. Cavitation-induced micro-mixing and enhanced interfacial mass transfer facilitate Au precursor transport and nucleation while suppressing particle growth and coalescence. A control experiment demonstrates that ultrasonic irradiation alone increases structural disorder without improving capacitive performance, indicating that the beneficial effect of ultrasound primarily arises from its role in controlling AuNP deposition. Quantitative TEM analysis shows that ultrasound-assisted deposition produces smaller and more uniformly distributed AuNPs (26.88 nm) than magnetic stirring (81.79 nm), which causes pronounced agglomeration. The optimized ACAu-20–60-ul electrode retains a high specific surface area of 2180 m^2^ g^−1^ and delivers 387F g^−1^ at 0.5 A g^−1^, representing a 66% enhancement over pristine AC700 (233F g^−1^). In a symmetric device, ACAu-20–60-ul achieves 160F g^−1^ at 0.5 A g^−1^ and 22.2 Wh kg^−1^ at 500 W kg^−1^, with 96.5% capacitance retention after 5000 cycles. These results demonstrate that acoustic cavitation effectively controls AuNP formation and dispersion, providing an effective sonochemical strategy for engineering high-performance biomass-derived carbon electrodes.

## 1 Introduction

The rapid depletion of fossil fuel resources and the increase in environmental concerns associated with greenhouse gas emissions have stimulated extensive research on sustainable and efficient energy storage systems [1]. In recent years, electrochemical energy storage devices have attracted considerable attention due to their capability to store and deliver electrical energy efficiently [2], [3]. Among various energy storage technologies, supercapacitors have emerged as promising candidates because of their high-power density, rapid charge–discharge capability, and long cycle life [4]. These advantages make supercapacitors particularly attractive for applications in portable electronics, hybrid electric vehicles, and renewable energy systems. However, despite these benefits, supercapacitors still suffer from relatively low energy density compared with conventional batteries, which limits their broader practical applications [5], [6]. Therefore, significant research efforts have been devoted to improving the electrochemical performance of supercapacitors through the development of advanced electrode materials [7], [8].

Recently, carbon materials including graphene [9], carbon nanotubes [10], carbon aerogel [11], and activated carbon [12] have been comprehensively researched to produce high-performance supercapacitor electrode. Among these carbon materials, activated carbon has been widely used as an electrode material in supercapacitors due to its high specific surface area, excellent chemical stability, and relatively low cost [7], [13]. The porous structure of activated carbon provides abundant active sites for electrolyte ion adsorption, which is essential for efficient charge storage in electric double-layer capacitors (EDLCs) [14]. Agricultural waste has been recognized as promising precursor for producing activated carbon [15], [16]. Rice-straw-derived soluble fractions offer a value-added pathway for converting abundant agricultural residues into functional carbon electrode materials for electrochemical energy storage [17], [18]. Despite the favorable properties of biomass-derived activated carbon, its electrochemical performance is still limited by relatively low electrical conductivity and insufficient electrochemical activity, thereby reducing the overall capacitance and rate capability of the electrode materials [19], [20]. To address these challenges, various strategies have been explored to enhance the electrochemical properties of activated carbon, including heteroatom doping, surface functionalization, and the incorporation of conductive nanomaterials [21], [22]. Among these approaches, the decoration of carbon materials with metal nanoparticles has shown to be an effective strategy for improving electrical conductivity and facilitating charge transfer at the electrode–electrolyte interface [23].

Gold nanoparticles (AuNPs) have attracted considerable interest owing to their distinctive electronic and surface properties, chemical stability, and tunable interfacial characteristics [24], [25]. Their particle size and morphology are strongly influenced by synthesis conditions [26], [27], which is particularly important for AuNP-modified carbon electrodes because nanoparticle dispersion can affect electrode–electrolyte interfacial behavior and electrochemical performance. Çiplak et al. found that the incorporation of AuNPs into carbon-based materials could enhance electron transport pathways, improve electrical conductivity, and promote faster electrochemical reactions [28]. Sharifi et al. reported that the composite of graphene quantum dots and AuNPs exhibited a high specific capacitance of 480F g^−1^ at current density of 0.5 A g^−1^ [29]. Ji et al. discussed that the performance of metal nanoparticle-decorated carbon materials strongly depended on the dispersion, size, and distribution of the nanoparticles on the carbon surface [30]. Ma et al. prepared AuNPs decorated carbon using conventional magnetic stirring and found that the AuNPs decorated carbon showed a high specific capacitance of 440F g^−1^ at current density of 0.5 A g^−1^ [31]. However, the magnetic stirring method often led to particle agglomeration and non-uniform distribution of nanoparticles, which could significantly reduce the effective surface area and limit the enhancement of electrochemical performance. Therefore, developing more efficient synthesis methods to achieve uniform nanoparticle dispersion and strong interaction between metal nanoparticles and carbon substrates remains an important challenge.

Ultrasonic irradiation induces acoustic cavitation through the formation, growth, and implosive collapse of bubbles in the liquid medium [32]. The collapse of cavitation bubbles generates localized physical effects, including shock waves, microjets, and intense micro-mixing, which can enhance mass transfer and reduce diffusion limitations at solid–liquid interfaces [33]. During AuNP deposition, these cavitation-induced effects may facilitate the transport of Au precursor species toward the activated carbon surface and promote more homogeneous nucleation [34], [35]. Enhanced local mixing may suppress particle growth and coalescence, resulting in smaller, well-dispersed AuNPs that facilitate interfacial charge transfer and enhance electrochemical performance. Recently, Tanaka et al. applied ultrasound irradiation in deposition of AuNPs on cathode material for Li ion batteries [36]. Compared with conventional magnetic stirring methods, ultrasound irradiation can facilitate better dispersion of metal precursors and promote more uniform nucleation of nanoparticles on the carbon surface, which are beneficial for improving electrochemical performance.

In this study, activated carbon derived from rice straw soluble was successfully synthesized and utilized as a carbon support for AuNPs decoration. Gold nanoparticles were deposited onto the activated carbon surface using an ultrasound irradiation method at a frequency of 200 kHz. The structural and morphological characteristics of the prepared materials were systematically investigated using various characterization techniques. Furthermore, the electrochemical performance of the electrodes was evaluated using cyclic voltammetry, galvanostatic charge–discharge, and electrochemical impedance spectroscopy to assess their potential application in supercapacitors.

## 2 Experimental

### 2.1 Materials

Rice straw (RS) obtained from Nakhon Ratchasima province, Thailand, was employed as the biomass precursor throughout this investigation. Prior to utilization, the as-received RS was subjected to mechanical size reduction through cutting operations to achieve a particle size of less than 100 µm. Potassium hydroxide (KOH, Kemaus) served as the chemical activating agent for the fabrication of porous carbon materials. The decoration of activated carbon with gold nanoparticles was accomplished using HAu(III)Cl_4_·4H_2_O (99.9%, Wako) as the Au precursor source. All chemical reagents were of analytical purity and were utilized directly without any further purification steps.

### 2.2 Preparation of activated carbon

The rice-straw soluble fraction was prepared by degradative solvent extraction (DSE), following our previously reported procedure [37] with additional experimental details provided here for reproducibility. Briefly, 60 g of rice straw was mixed with 3000 mL of A-150 solvent, corresponding to a biomass-to-solvent ratio of 1:50 (g mL^−1^), and loaded into a PARR autoclave reactor. The mixture was heated to 350 °C and maintained at this temperature for 60 min under a pressure of approximately 80–100 bar. The resulting rice-straw soluble fraction was recovered with an extraction yield of approximately 25–30 wt%. The resulting soluble fraction was then carbonized at 700 °C for 1 h under a nitrogen atmosphere at a heating rate of 10 °C min^−1^. The char obtained was subsequently activated with potassium hydroxide (KOH) at a carbon-to-KOH mass ratio of 1:3 at 700 °C for 1 h under nitrogen flow at the same heating rate. After activation, the products were washed sequentially with 0.1 M HCl solution and deionized water until neutral pH was reached, ensuring complete removal of residual inorganic impurities. The resulting samples were dried at 110 °C for 24 h. The activated carbon product thus prepared was designated as AC700.

### 2.3 Synthesis of Au decorated activated carbon

Gold precursor solutions were prepared by dissolving hydrogen tetrachloroaurate (III) tetrahydrate (HAuCl_4_·4H_2_O) at varying loadings (20 and 30 mg) in a binary solvent system comprising 2 mL of 2-propanol and 100 mL of deionized (DI) water, where 2-propanol was first added to facilitate precursor dissolution and improve dispersion prior to the addition of DI water. Separately, 0.5 g of AC700 was pre-wetted with 2 mL of ethanol to enhance surface wettability and promote uniform interfacial contact with the Au precursor solution. The prepared gold solution was then added into the carbon suspension, and the combined mixture was subsequently degassed by purging with argon gas for 30 min to remove dissolved oxygen and prevent unwanted oxidation reactions from occurring during the deposition process. For ultrasound-assisted deposition, the suspension was irradiated using a high-frequency ultrasonic processor (KAIJO, Quava Mini) operating at 200 kHz and 100 W for durations of 20, 60, and 120 min. The ultrasonic power reached to the solution (100 mL) was estimated to be 7.4 W using a calorimetry method. For comparative purposes, a conventional magnetic stirring approach was also employed, in which the mixture was agitated at 300 rpm at room temperature for 60 min. Following deposition, the samples were vacuum-filtered, washed thoroughly with DI water to remove residual ionic species, and dried at 110 °C overnight. The dried samples were then subjected to sequential thermal treatment: pyrolysis at 700 °C for 1 h under nitrogen atmosphere (heating rate: 10 °C min^−1^), followed by oxidation at 300 °C for 30 min in air at the same heating rate. For each investigated Au-deposition condition, two independent synthesis batches were prepared. The products obtained from the two batches were subsequently combined and homogenized prior to electrode fabrication. The resulting Au-decorated activated carbon samples were designated as ACAu-X-Y-Z, where X denoted the Au precursor loading (mg), Y represented the treatment duration (min), and Z indicated the deposition method (“ul” for ultrasonic irradiation and “st” for magnetic stirring). The ultrasound treated activated carbons without Au loading were designated as AC-0-X-ul, where X represented the treatment duration (min). The overall synthesis procedure is schematically illustrated in Fig. 1.

**Fig. 1:**
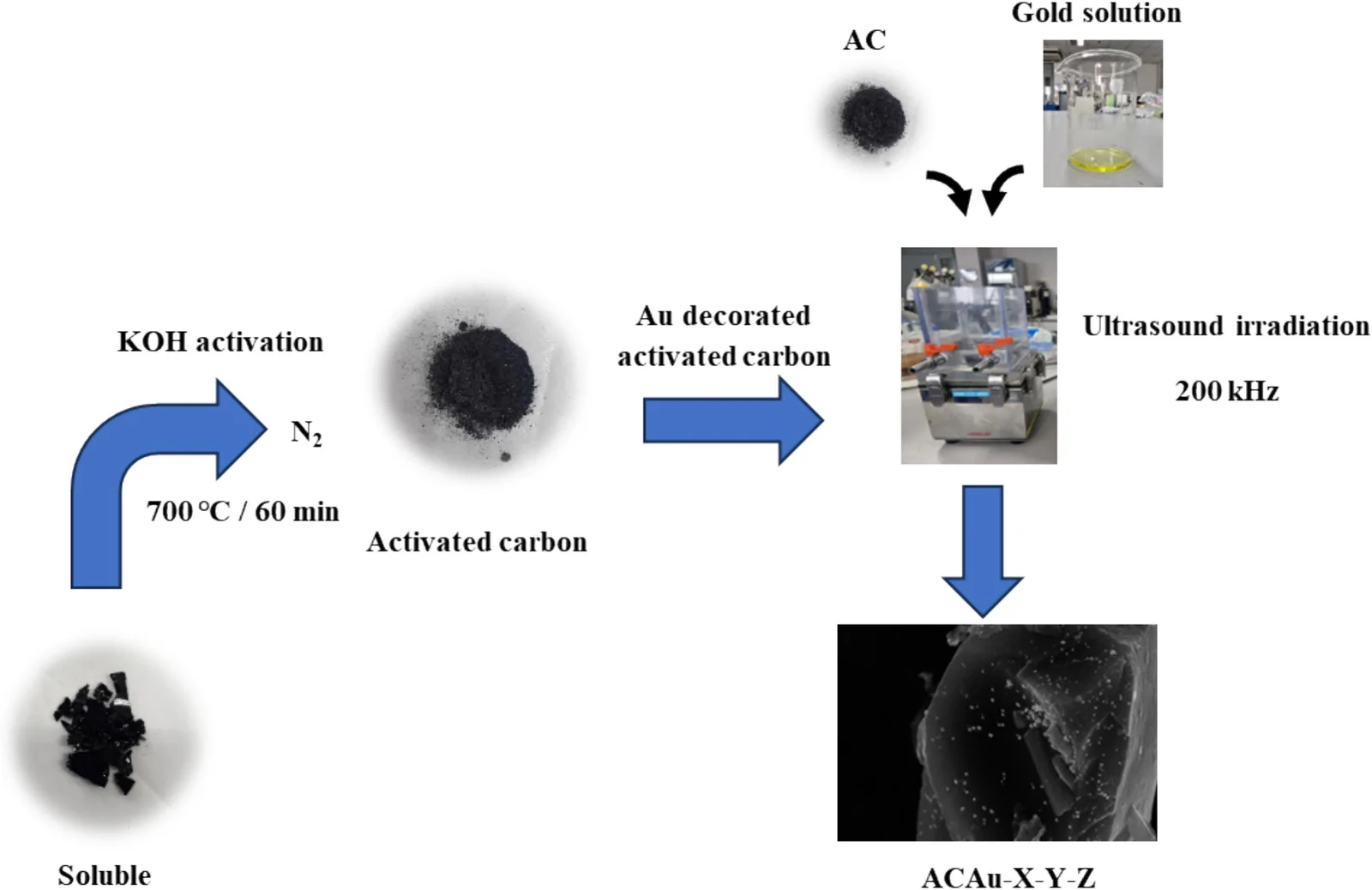
Schematic diagram of the synthesis for AuNPs decorated activated carbon derived from rice straw soluble fraction.

### 2.4 Surface characterization of carbon materials

The microstructural features and morphological characteristics of the as-prepared samples were examined by transmission electron microscopy (TEM, JEOL JEM-2100 Plus). Bulk elemental composition (C, H, and N contents) was quantified using a CHN elemental analyzer (J-Science Lab, CHN JM10). Crystal structure characterization was conducted by X-ray diffraction (XRD, Bruker D2 Phaser) utilizing Cu Kα radiation (λ = 1.5418 Å) at an accelerating voltage of 40 kV and a tube current of 100 mA. The extent of structural disorder and graphitic character of the carbon materials were probed by Raman spectroscopy employing a micro-Raman spectrometer (Horiba, Xplora™ Plus) with a 514 nm argon ion laser as the excitation source. Textural characteristics, encompassing specific surface area and pore size distribution, were determined from nitrogen adsorption–desorption isotherms acquired at 77 K on a volumetric surface area analyzer (BEL Japan, BELSORP-mini II). The specific surface area was computed by applying the Brunauer–Emmett–Teller (BET) model, whereas pore size distribution was extracted from the adsorption branch of the isotherm. Surface elemental composition and chemical bonding states were investigated by X-ray photoelectron spectroscopy (XPS, Kratos Axis Supra-8, Shimadzu). The Au content of the decorated samples was quantitatively assessed by inductively coupled plasma atomic emission spectroscopy (ICP-AES, Shimadzu ICPE-9820), following complete dissolution of the samples in a microwave digestion system (Milestone, Ultrawave) to guarantee total recovery of Au species.

### 2.5 Electrochemical measurements

The electrochemical performance of the as-fabricated electrodes was evaluated in a three-electrode cell setup employing 6 M KOH aqueous solution as the electrolyte, a platinum mesh as the counter electrode, and an Ag/AgCl electrode as the reference electrode. Working electrodes were prepared by formulating a homogeneous slurry composed of the active material, carbon black, and polyvinylidene fluoride (PVDF) in a weight ratio of 8:1:1, which was subsequently cast onto nickel foam current collectors (1 × 1 cm^2^). The coated electrodes were then dried at 100 °C for 12 h and compressed under 30 MPa, yielding an active material mass loading of approximately 5 mg cm^−2^. For each sample, two independently prepared electrodes were fabricated from the combined material. CV and GCD measurements were repeated five times for each electrode under identical conditions, and the average values were used for analysis. All electrochemical measurements were carried out on a potentiostat/galvanostat (Metrohm Autolab PGSTAT302N) controlled via NOVA 1.11 software. In the three-electrode system, CV measurements were conducted over a potential window of − 0.2 to − 1.2 V at scan rates of 5 to 100 mV s^−1^, and GCD measurements were performed at current densities ranging from 0.5 to 50 A g^−1^. Long-term cycling stability was evaluated over 5000 consecutive charge–discharge cycles. To assess practical device performance, symmetric supercapacitors were assembled in a two-electrode configuration using AC700, ACAu-20–60-ul, and ACAu-20–60-st as electrode materials. The electrodes were prepared following the same procedure described above, with copper foil discs (11 mm diameter) as current collectors and filter paper as the separator. CV and GCD measurements for the two-electrode devices were conducted over a voltage window of − 0.5 to 0.5 V at scan rates of 5 to 100 mV s^−1^ and current densities of 0.5 to 50 A g^−1^, respectively. EIS measurements were performed at open-circuit potential with an AC perturbation of 5 mV over a frequency range of 10 mHz to 100 kHz. The specific capacitance of the three-electrode system (C_m_, F g^−1^) was calculated from GCD discharge profiles using Equation (1), while the device capacitance of the symmetric supercapacitor was obtained from Equation (2).

(1) $$C_{m}=\frac{I\Delta t}{m\Delta V}$$

(2) $$C_{m}=2\frac{I\Delta t}{m\Delta V}$$

In Equation (1), *I* denotes the applied discharge current (A), *Δt* the discharge duration (s), *ΔV* the working potential window (V), and *m* the mass of electroactive material on the electrode (g). It should be noted that in the three-electrode system, m refers only to the mass of active material on the working electrode, whereas in the symmetric two-electrode device, *m* represents the total mass of active material on both electrodes combined. The energy density (E, Wh kg^−1^) and power density (P, W kg^−1^) of the symmetric supercapacitor device were then derived from the GCD discharge profiles using Equations (3), (4), respectively.

(3) $$E=\frac{C_{m}({\Delta V)}^{2}}{2\times 3.6}$$

(4) $$P=\frac{3600\times E}{\Delta t}$$

In Equations (3), (4), *C_m_* refers to the gravimetric specific capacitance (F g^−1^) extracted from Equation (1), *ΔV* the applied operational voltage window (V), and *Δt* the complete discharge duration (s) of the assembled symmetric supercapacitor device.

## 3 Result and discussion

### 3.1 Effect of ultrasound irradiation time on the physicochemical and electrochemical properties of AC700

To isolate the intrinsic effect of ultrasonic irradiation on the carbon framework, pristine AC700 was subjected to ultrasonic treatment at varying durations (20, 60, and 120 min) in the absence of any Au precursor. This control experiment was designed to determine whether ultrasound alone could induce measurable changes in the structural and electrochemical properties of the carbon material prior to metal nanoparticle incorporation. The corresponding results are presented in Fig. 2 and Table 1. The N_2_ adsorption–desorption isotherms (Fig. 2a) of all samples exhibit type I characteristics, evidenced by a sharp adsorption uptake at low relative pressures (P/P_0_ < 0.1), which is indicative of a predominantly microporous structure [38]. The isotherm profiles of the ultrasound-treated samples remain virtually indistinguishable from that of pristine AC700, suggesting that the overall pore architecture is well preserved following ultrasonic treatment. This observation is corroborated by the textural parameters summarized in Table 1. The BET specific surface area (S_BET_) varies only marginally, from 2142 m^2^ g^−1^ for AC700 to 2179, 2165, and 2172 m^2^ g^−1^ for samples treated at 20, 60, and 120 min, respectively. Similarly, the total pore volume (V_total_) and micropore volume (V_mic_) show negligible differences across all samples, confirming that ultrasonic irradiation does not appreciably alter the porous architecture of the carbon matrix. The pore size distribution curves (Fig. 2b) further support these findings, as all samples display analogous micropore-dominated profiles, although AC-0–120-ul exhibits a marginally elevated differential pore volume at the smallest micropore range (∼1.7 nm). Raman spectroscopy (Fig. 2c) reveals a systematic increase in the I_D_/I_G_ ratio from 0.83 for pristine AC700 to 0.86, 0.88, and 0.89 for samples treated at 20, 60, and 120 min, respectively, indicating a progressive accumulation of structural defects and increasing lattice disorder within the carbon framework with prolonged ultrasonic exposure. This behavior is attributed to acoustic cavitation, wherein the violent collapse of transient microbubbles generates localized extreme conditions — including high temperatures, shockwaves, and microjets — capable of disrupting sp^2^ carbon domains at the nanoscale [39], [40]. The cumulative nature of these high-energy events with increasing irradiation time accounts for the gradual rise in defect density observed. The XRD patterns (Fig. 2d) of all samples display a broad, ill-defined feature in the 20–30° region attributable to the (002) reflection of turbostratic carbon [41], along with a distinct diffraction peak at approximately 43° corresponding to the (1 0 0) reflection. Only negligible variations in peak characteristics are observed among the samples, indicating that the long-range graphitic ordering remains essentially unaffected by ultrasonic treatment. This implies that the structural modifications induced by ultrasound are highly localized and do not perturb the bulk crystalline framework, a finding consistent with the Raman spectroscopic results. The electrochemical response of the samples was assessed via CV and GCD measurements (Fig. 2e and 2f). All electrodes display quasi-rectangular CV profiles, characteristic of electric double-layer capacitive (EDLC) behavior, confirming that charge storage is predominantly governed by reversible ion adsorption and desorption at the electrode–electrolyte interface. Nevertheless, a progressive reduction in the enclosed CV area is observed with increasing ultrasonic irradiation duration, with AC700 exhibiting the largest area, followed by AC-0–20-ul, AC-0–60-ul, and AC-0–120-ul, indicating a gradual deterioration in capacitive performance. This trend is consistently reflected in the GCD profiles (Fig. 2f), where AC700 displays the longest discharge time, while AC-0–20-ul, AC-0–60-ul, and AC-0–120-ul exhibit comparably shorter and closely similar discharge times. The specific capacitance values calculated at a current density of 0.5 A g^−1^ follow the same trend, decreasing from 233F g^−1^ for pristine AC700 to 205, 194, and 193F g^−1^ for AC-0–20-ul, AC-0–60-ul, and AC-0–120-ul, respectively. These results suggest that even short ultrasonic exposure leads to a discernible reduction in charge storage capacity, with prolonged treatment yielding only marginal additional degradation. Despite the negligible changes in surface area and pore structure, the decline in electrochemical performance is attributed to the increased structural disorder introduced by ultrasonic irradiation. The generation of excessive defect sites may disrupt the continuity of the conductive carbon network, thereby impeding electron transport. In addition, surface irregularities along the pore walls arising from defect formation could elevate ion transport resistance within the microporous channels, compromising the efficiency of charge accumulation. It is noteworthy that not all defect types contribute favorably to capacitive performance; while a moderate defect density can provide supplementary active sites for charge storage, an excess of disordered domains may instead degrade charge transport pathways and increase internal resistance [42]. Despite the negligible changes in specific surface area and pore structure, Raman analysis showed a progressive increase in structural disorder with increasing ultrasonic irradiation time, accompanied by a decrease in specific capacitance from 233F g^−1^ for pristine AC700 to 205, 194, and 193F g^−1^ after 20, 60, and 120 min of treatment, respectively. This correlation suggests that ultrasound-induced structural disorder may adversely affect electron transport within the carbon framework, thereby contributing to the decrease in capacitance. These results indicate that ultrasonic irradiation alone does not improve the capacitive performance of AC700. Although acoustic cavitation can generate intense local mechanical effects capable of modifying carbon surfaces and structures [43], the present results demonstrate that ultrasonic irradiation alone does not improve the capacitive performance of AC700.

**Fig. 2:**
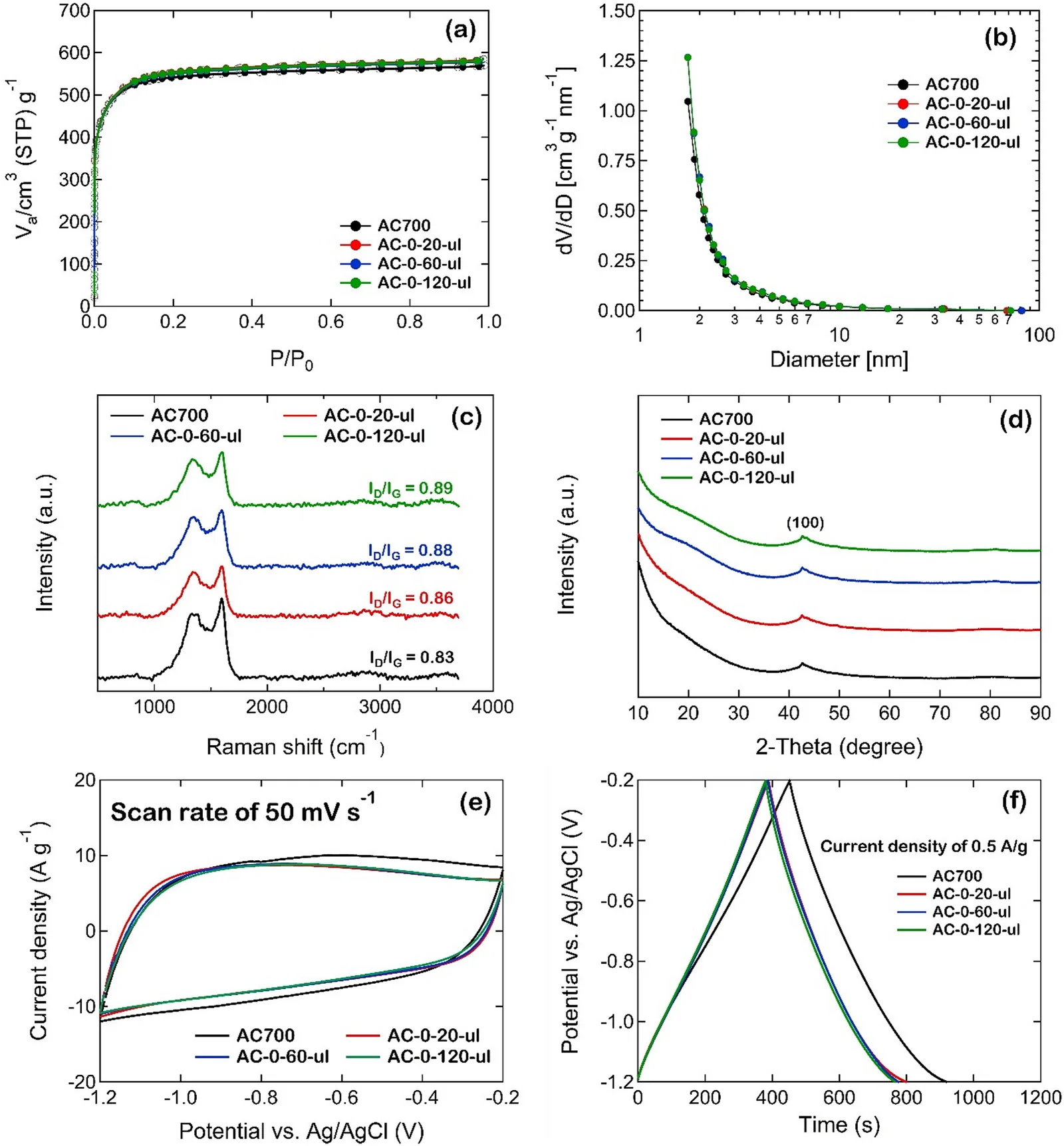
(a) N_2_ adsorption–desorption isotherms, (b) pore size distribution curves, (c) Raman spectra, (d) X-ray diffraction patterns, (e) CV curves at a scan rate of 50 mV s^−1^, and (f) GCD curves at a current density of 0.5 A g^−1^ of AC700, AC-0–20-ul, AC-0–60-ul, and AC-0–120-ul.

**Table 1:** Ultimate analysis and specific surface area of various derived carbon.

Samples	Ultimate Analysis [wt%, d.a.f.]				Amount of Au (mg kg^−1^)	S_BET_ (m^2^g^−1^)	V _total_^a^ (cm^3^ g^−1^)	V _mic_^b^ (cm^3^g^−1^)	V *_meso_*_-macro_^c^ (cm^3^ g^−1^)	D _avg_ (nm)
	C	H	N	O (diff)						
AC700	91.9	0.3	0.7	7.1	−	2142	0.88	0.87	0.01	1.64
AC-0–20-ul	93.6	0.7	0.8	4.9	−	2179	0.90	0.89	0.01	1.65
AC-0–60-ul	92.7	0.5	0.6	6.2	−	2165	0.90	0.89	0.01	1.67
AC-0–120-ul	92.2	0.8	0.6	6.4	−	2172	0.90	0.89	0.01	1.66
ACAu-20–20-ul	90.6	0.3	0.3	8.8	n.d.	2172	0.90	0.89	0.01	1.67
ACAu-20–60-ul	90.3	0.2	0.5	9.0	2019	2180	0.91	0.89	0.02	1.67
ACAu-20–120-ul	90.6	0.3	0.4	8.7	1005	2142	0.89	0.88	0.01	1.67
ACAu-30–120-ul	90.9	0.2	0.5	8.4	932	2099	0.88	0.86	0.02	1.67
ACAu-20–60-st	92.6	0.2	0.5	6.7	1182	2056	0.88	0.84	0.04	1.71

n.d. = Not determined.

a Total pore volume at P/P_0_ = 0.99.

b t-plot micropore volume.

c V_meso – macro_ = V_total_ – V_micro_.

### 3.2 Physicochemical and structural properties of Au-decorated activated carbon

The morphology and spatial distribution of AuNPs on the carbon matrix were examined by TEM, HRTEM, and STEM–EDS analyses, with representative images presented in Fig. 3. Pristine AC700 (Fig. 3a) exhibits a thin, sheet-like carbon morphology with well-defined edges, consistent with a turbostratic and porous carbon framework. No particulate features are noticeable, confirming the absence of metallic species prior to Au deposition. Following Au deposition via ultrasonic irradiation, the ACAu-20–60-ul sample (Fig. 3b and 3c) exhibits a high density of nanoparticles distributed across the carbon surface. At low magnification (Fig. 3b), the nanoparticles appear homogeneously dispersed with no remarkable aggregation, indicating effective nucleation throughout the carbon matrix. At higher magnification (Fig. 3c), the particles are relatively small and evenly distributed, with diameters estimated in the range of approximately 12–68 nm, suggesting that ultrasonic irradiation promotes controlled nucleation while suppressing particle growth. In contrast, the ACAu-20–60-st sample prepared via conventional magnetic stirring (Fig. 3d and 3e) displays a markedly different particle morphology. Extensive clustering and severe agglomeration of AuNPs are observed at low magnification (Fig. 3d), where particles coalesce into irregular aggregates. At higher magnification (Fig. 3e), significantly larger particle sizes are evident, indicating that conventional stirring provides insufficient energy for uniform nucleation and instead favors uncontrolled particle growth and coalescence. To quantitatively evaluate the effect of the deposition method on AuNP size, particle-size distributions were determined from 150 nanoparticles for each sample, as shown in Fig. 3f and 3 g. ACAu-20–60-ul exhibited a smaller mean particle size of 26.88 nm with a median of 23.33 nm and a relatively narrow size distribution range of 11.69 to 67.80 nm. In contrast, ACAu-20–60-st showed a substantially larger mean particle size of 81.79 nm and median of 63.56 nm, with a broad distribution ranging from 12.66 to 342.39 nm. These quantitative results are consistent with the TEM observations and indicate that ultrasound-assisted deposition favors smaller and more uniformly distributed AuNPs, whereas magnetic stirring results in a broader particle-size distribution and greater particle aggregation. HRTEM analysis of ACAu-20–60-ul (Fig. 3h) revealed well-resolved lattice fringes with a measured d-spacing of approximately 0.24 nm, which can be assigned to the (1 1 1) crystallographic plane of face-centered cubic (fcc) Au [31]. This observation provides additional structural evidence for the presence of crystalline AuNPs on the activated carbon support. Complementary STEM–EDS analysis (Fig. 3i–3 k) provides further confirmation of successful Au deposition. The STEM image (Fig. 3i) reveals bright contrast spots attributable to the presence of high atomic number elements, consistent with AuNPs anchored on the lighter carbon support. Elemental mapping confirms the distribution of carbon (Fig. 3j) and gold (Fig. 3k) within the analyzed region. The Au signal is predominantly co-localized with the carbon-rich domains, confirming successful anchoring of AuNPs onto the carbon surface, while regions of lower carbon density exhibit correspondingly weaker Au signals. Collectively, the TEM and EDS results show that ultrasound-assisted deposition produces smaller and more uniformly distributed AuNPs than magnetic stirring. This difference is consistent with the enhanced mixing and mass-transfer effects commonly associated with acoustic cavitation [44], which may promote more homogeneous nanoparticle formation and reduce particle coalescence. These morphological differences may influence the electrochemical behavior by affecting AuNP dispersion and pore accessibility.

**Fig. 3:**
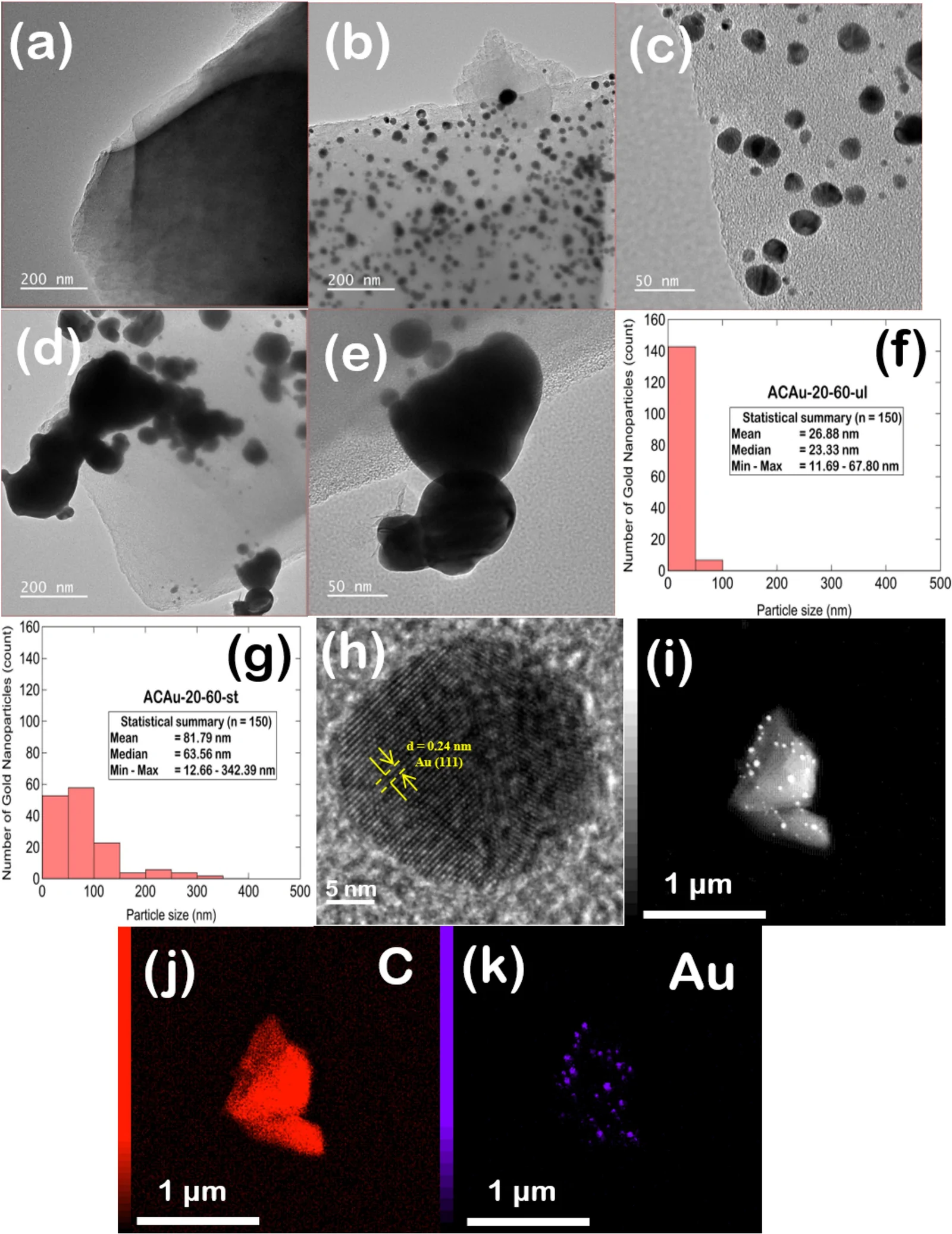
TEM images of (a) pristine AC700, (b, c) ACAu-20–60-ul at low and high magnification, and (d, e) ACAu-20–60-st at low and high magnification, particle-size distributions of (f) ACAu-20–60-ul and (g) ACAu-20–60-st, (h) HRTEM image of ACAu-20–60-ul showing lattice fringes of Au nanoparticles; (i) STEM image and corresponding EDS elemental mapping of (j) carbon (C) and (k) gold (Au) for ACAu-20–60-ul.

The physicochemical properties of the Au-decorated activated carbon samples were systematically characterized by N_2_ adsorption–desorption analysis, Raman spectroscopy, X-ray diffraction, and elemental analysis, with the results presented in Fig. 4a–4d and Table 1. The N_2_ adsorption–desorption isotherms (Fig. 4a) of all samples display type I characteristics, with a sharp adsorption uptake at low relative pressures (P/P_0_ < 0.1), indicative of a predominantly microporous structure. The isotherm profiles of the Au-decorated samples remained largely unchanged relative to that of pristine AC700, suggesting that Au incorporation does not substantially perturb the underlying pore architecture. This is further supported by the textural parameters in Table 1, where the S_BET_ of AC700 (2142 m^2^ g^−1^) varies only slightly upon modification. Notably, ACAu-20–60-ul retains a comparable S_BET_ of 2180 m^2^ g^−1^, demonstrating that ultrasound-assisted deposition effectively preserves the high surface area of the carbon support. In contrast, marginal reductions in S_BET_ are observed for ACAu-20–120-ul (2142 m^2^ g^−1^), ACAu-30–120-ul (2099 m^2^ g^−1^) and ACAu-20–60-st (2056 m^2^ g^−1^), which may be ascribed to partial pore blockage resulting from AuNPs aggregation or over-deposition [45]. Similarly, the pore size distribution curves (Fig. 4b) show that all samples retain a predominantly microporous structure with pore diameters centered at approximately 2 nm. These results indicate that the porous architecture is largely preserved following Au incorporation. Raman spectra (Fig. 4c) of all samples exhibit the characteristic D band (∼1340 cm^−1^) and G band (∼1590 cm^−1^), corresponding to defect-induced disordered carbon and in-plane stretching vibrations of sp^2^ graphitic domains, respectively. The I_D_/I_G_ ratio increases systematically from 0.83 for pristine AC700 to 0.87, 0.89,0.90, 0.90, and 0.89 for ACAu-20–20-ul, ACAu-20–60-ul, ACAu-20–120-ul, ACAu-30–120-ul, and ACAu-20–60-st, respectively, indicating that Au deposition introduces additional structural disorder into the carbon framework regardless of the deposition method employed. The highest I_D_/I_G_ value (0.90) recorded for ACAu-20–120-ul, and ACAu-30–120-ul suggests that prolonged ultrasonic irradiation at higher Au loading induces greater defect accumulation. It is noteworthy that ACAu-20–60-ul and ACAu-20–60-st exhibit identical I_D_/I_G_ values (0.89), implying that comparable levels of structural disorder are introduced by both methods, albeit through distinctly different mechanisms — cavitation-driven disruption in the case of ultrasound, and thermally driven surface interactions in the case of stirring. The XRD patterns (Fig. 4d) of all samples display a broad, ill-defined feature in the 20–30° region attributable to the (002) reflection of turbostratic carbon [41], along with a weak peak at approximately 43° corresponding to the (100) reflection, confirming the amorphous nature of the carbon matrix. Upon Au incorporation, additional diffraction features were observed at approximately 38°, 44°, 64°, and 80°, assignable to the (111), (200), (220), and (222) reflections of face-centered cubic (fcc) Au [46], respectively. Elemental composition and textural parameters of all samples are summarized in Table 1. The ultimate analysis of pristine AC700 revealed a high carbon content of 91.9 wt% (d.a.f.), with minor contributions from hydrogen (0.3 wt%), nitrogen (0.7 wt%), and oxygen (7.1 wt%), consistent with a carbon-rich framework derived from biomass precursors. Upon ultrasonic treatment without Au precursor (AC-0–20-ul, AC-0–60-ul, and AC-0–120-ul), the carbon content varied slightly in the range of 92.2–93.6 wt%, while oxygen content decreased marginally to 4.9–6.4 wt%, suggesting minor surface compositional changes induced by ultrasonic irradiation alone. For Au-decorated samples, the carbon content remained comparable at approximately 90.3–92.6 wt%, indicating that Au incorporation does not substantially alter the bulk elemental composition of the carbon framework. The Au content quantified by ICP-AES was determined to be 2019, 1005, 932, and 1182 mg kg^−1^ for ACAu-20–60-ul, ACAu-20–120-ul, ACAu-30–120-ul, and ACAu-20–60-st, respectively. Among the ultrasound-treated samples, ACAu-20–60-ul exhibited the highest measured Au content (2019 mg kg^−1^, approximately 0.202 wt%), whereas ACAu-20–120-ul and ACAu-30–120-ul showed lower values of 1005 and 932 mg kg^−1^, respectively. These results indicate that increasing the precursor loading or extending the irradiation time did not proportionally increase the retained Au content under the investigated conditions. ACAu-20–60-st exhibited an Au content of 1182 mg kg^−1^, lower than that of ACAu-20–60-ul. Despite Au incorporation, the high specific surface area and well-preserved microporous structure were retained, as evidenced by the BET and pore size distribution analyses, indicating that Au incorporation did not cause substantial pore occlusion. Taken together, these results indicate that ultrasound-assisted deposition enables Au incorporation while preserving the intrinsic porous framework of the activated carbon support. In contrast, the stirring-prepared sample showed minor differences in textural properties, together with the larger particle size and greater aggregation observed by TEM. The retention of high surface area and microporosity, together with the more uniform AuNP dispersion observed for the ultrasound-treated sample, may contribute to its improved electrochemical performance.

**Fig. 4:**
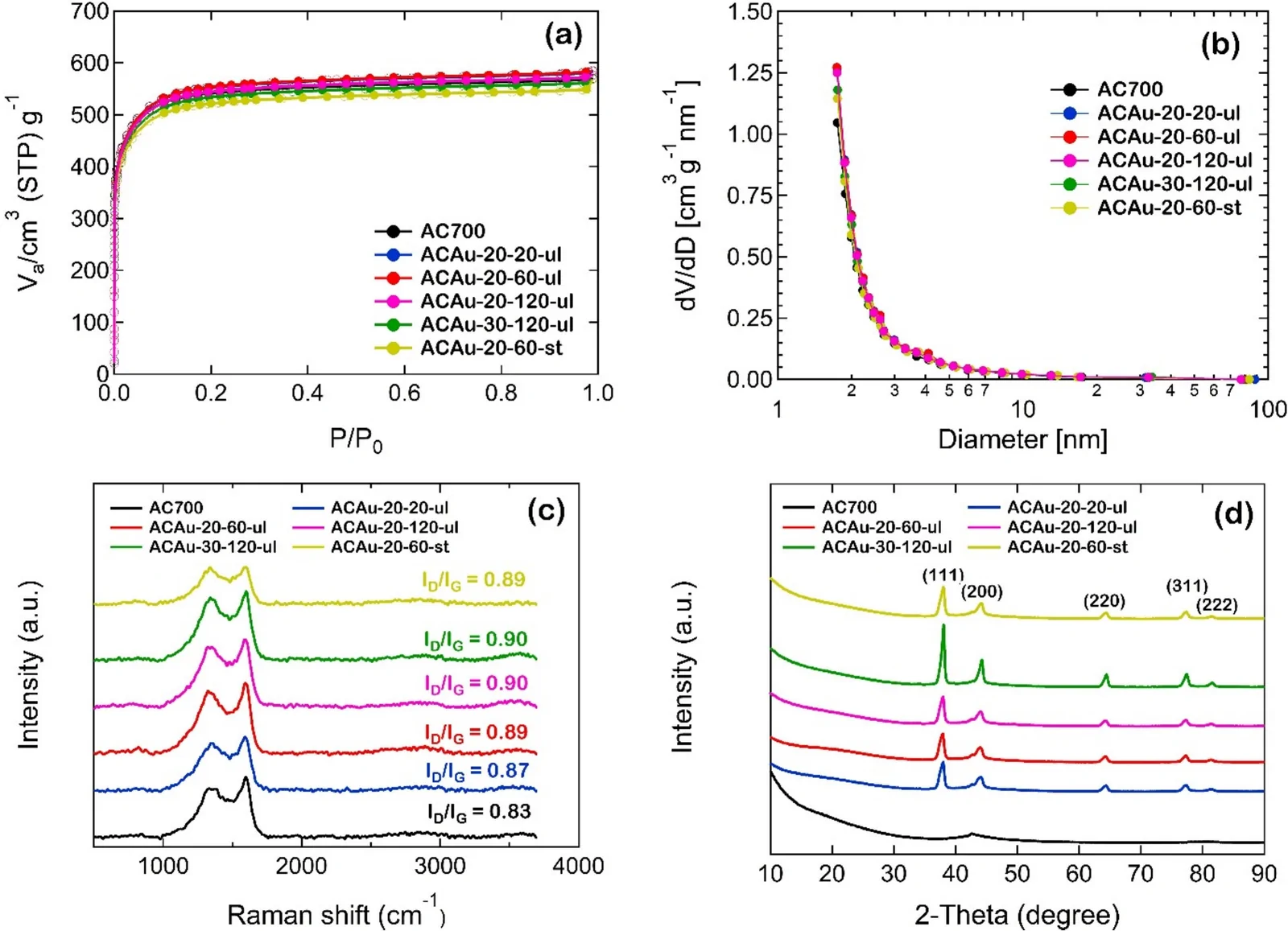
(a) N_2_ adsorption–desorption isotherms, (b) pore size distribution curves, (c) Raman spectra, and (d) X-ray diffraction patterns of AC700, ACAu-20–20-ul, ACAu-20–60-ul, ACAu-20–120-ul, ACAu-30–120-ul, and ACAu-20–60-st.

The surface chemical composition and elemental binding states of the prepared samples were investigated by X-ray photoelectron spectroscopy (XPS), with the results presented in Fig. 5. The survey spectra (Fig. 5a) of AC700, ACAu-20–60-ul, ACAu-20–120-ul, and ACAu-20–60-st display prominent C 1 s and O 1 s signals for all samples, confirming that the carbon framework remains the dominant surface constituent following modification. Notably, distinct Au 4f signals are detected exclusively in the Au-decorated samples, with no corresponding peak observed for pristine AC700, unambiguously confirming the successful incorporation of AuNPs onto the carbon surface. The Au 4f signal intensity in ACAu-20–60-ul is visibly more prominent than that of ACAu-20–60-st, indicating a higher surface concentration and more effective dispersion of Au species achieved under ultrasound-assisted deposition conditions. This observation is in good agreement with the ICP-AES results, where ACAu-20–60-ul exhibits a higher Au content than its stirring-prepared counterpart. To further elucidate the chemical state of the deposited Au, high-resolution Au 4f spectra were acquired and deconvoluted for both samples, as presented in Fig. 5b-5d. For ACAu-20–60-ul (Fig. 5b), two well-resolved peaks are observed at binding energies of approximately 84.0 and 87.7 eV, assigned to the Au 4f_7/2_ and Au 4f_5/2_ spin–orbit components, respectively. For ACAu-20–120-ul (Fig. 5c), Au 4f_7/2_ and Au 4f_5/2_ peaks are observed at approximately 84.0 and 88.0 eV, respectively. Similarly, ACAu-20–60-st (Fig. 5d) exhibits Au 4f_7/2_ and Au 4f_5/2_ peaks at approximately 84.0 and 88.0 eV, respectively. The Au 4f_7/2_ binding energies of both samples are consistent with the reported value for metallic Au^0^ [47], confirming that the deposited AuNPs predominantly exist in the zero-valent state. The absence of discernible peak shifts toward higher binding energies indicates that oxidized Au species (Au^+^ or Au^3+^) are negligible in both samples. The relative peak area ratios of Au 4f_7/2_ to Au 4f_5/2_ are determined to be 55.6%: 44.4%, 56.6%: 43.4%, and 56.5%: 43.5% for ACAu-20–60-ul, ACAu-20–120-ul, and ACAu-20–60-st, respectively. These values are in close agreement with the theoretical spin–orbit branching ratio of Au 4f (4:3, corresponding to 57.1%: 42.9%), validating the reliability of the peak deconvolution procedure [48]. The comparable Au 4f binding energies and peak area ratios observed for both samples indicate that the deposition method does not alter the intrinsic chemical state of Au, but rather governs its spatial distribution and degree of surface exposure on the carbon matrix. The presence of metallic Au^0^ is particularly advantageous for electrochemical energy storage applications, as zero-valent Au exhibits high electrical conductivity and facilitates interfacial charge transfer at the electrode–electrolyte interface. In conjunction with high specific surface area and well-preserved microporous architecture of the carbon support, the uniformly dispersed AuNPs are expected to provide supplementary conductive pathways while preserving the ion transport channels essential for efficient capacitive charge storage. Collectively, the XPS results confirm that ultrasound-assisted deposition achieves more effective surface incorporation of metallic AuNPs relative to conventional stirring, while maintaining the chemical integrity of the underlying carbon framework. The superior surface dispersion and enhanced accessibility of Au species in ACAu-20–60-ul are anticipated to contribute significantly to its improved electrochemical performance.

**Fig. 5:**
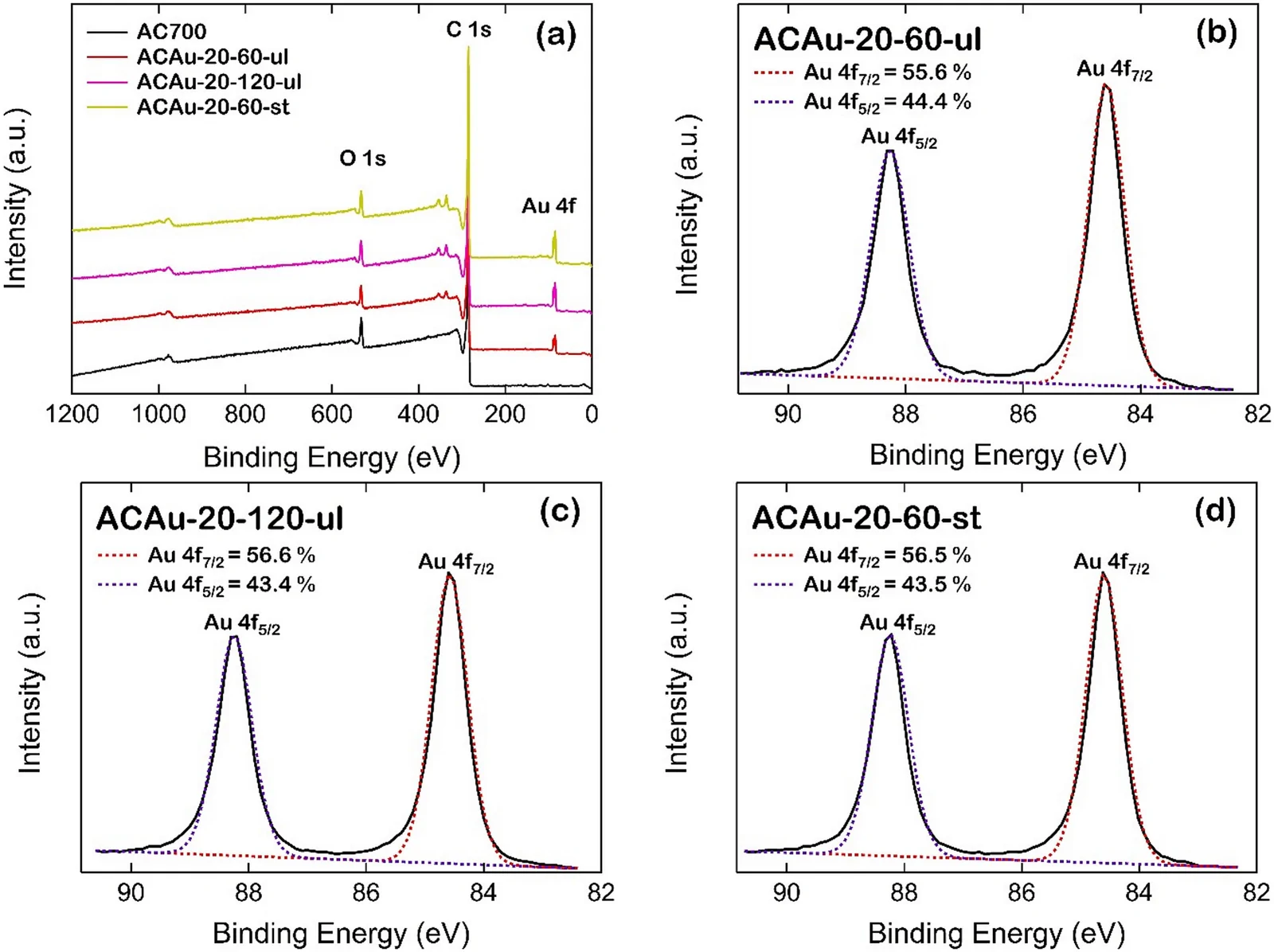
(a) XPS survey spectra of AC700, ACAu-20–60-ul, ACAu-20–120-ul and ACAu-20–60-st; high-resolution Au 4f spectra of (b) ACAu-20–60-ul, (c) ACAu-20–120-ul, and (d) ACAu-20–60-st.

### 3.3 Electrochemical properties of Au-decorated activated carbon in a three-electrode configuration

The electrochemical characteristics of the prepared electrode materials were assessed through cyclic voltammetry (CV) and galvanostatic charge–discharge (GCD) measurements conducted in a three-electrode cell setup, with the corresponding results illustrated in Fig. 6. The CV profiles recorded for all samples (Fig. 6a–6e) display nearly rectangular shapes devoid of any prominent redox peaks, establishing that the charge storage mechanism is principally dominated by electric double-layer capacitance (EDLC) originating from reversible electrolyte ion adsorption and desorption processes occurring at the electrode–electrolyte interface. The CV curve of pristine AC700 (Fig. 6a) displays a typical rectangular shape, however, its enclosed area is smaller than those of the Au-decorated counterparts. Upon Au incorporation, a pronounced enlargement of the CV area is observed, most notably for ACAu-20–60-ul (Fig. 6c), indicating a substantial enhancement in charge storage capability. This improvement is attributed to the introduction of well-dispersed conductive AuNPs, which facilitate electron transport, reduce charge transfer resistance, and improve the utilization efficiency of the microporous carbon surface for ion adsorption. A direct comparison of CV curves recorded at a scan rate of 50 mV s^−1^ (Fig. 6k) more clearly illustrates the performance distinctions among the samples. ACAu-20–60-ul exhibits the largest enclosed CV area, indicating the highest capacitive current response. ACAu-30–120-ul, ACAu-20–20-ul and ACAu-20–60-st display comparable CV areas, while AC700 exhibits the smallest area. This ordering demonstrates that ultrasound-assisted deposition generally outperforms conventional stirring in enhancing electrochemical performance, consistent with the superior AuNPs dispersion observed by TEM analysis. The GCD profiles (Fig. 6f–6j) of all samples display nearly symmetric triangular shapes, indicative of good electrochemical reversibility and high coulombic efficiency characteristic of EDLC behavior. A slight curvature at the onset of the discharge branch is observed for all samples, suggesting the presence of a minor internal resistance component, which is reduced upon Au incorporation. A direct comparison of GCD curves at a current density of 0.5 A g^−1^ (Fig. 6l) reveals that ACAu-20 60-ul exhibits the longest discharge time (∼1550 s), followed by ACAu-30–120-ul and ACAu-20–20-ul (∼1200 s), ACAu-20–60-st (∼1150 s), and AC700 (∼900 s), confirming the superior charge storage capacity of the ultrasound-optimized sample. The specific capacitance values derived from GCD discharge curves at 0.5 A g^−1^ are compiled in Table 2. Pristine AC700 delivers a capacitance of 233F g^−1^, whereas ACAu-20–20-ul, ACAu-20–60-ul, ACAu-30–120-ul, and ACAu-20–60-st achieve capacitances of 320, 387, 325, and 292F g^−1^, respectively. ACAu-20–60-ul attains the highest specific capacitance of 387F g^−1^, representing an enhancement of approximately 66% relative to pristine AC700. The homogeneous distribution of nanosized Au particles under ultrasonic conditions is believed to establish a more continuous conductive network across the carbon surface, thereby shortening electron transport pathways and improving rate capability [31], [49]. This remarkable improvement is ascribed to the synergistic combination of well-dispersed AuNPs and the preserved microporous carbon framework. In contrast, ACAu-20–60-st exhibits a lower specific capacitance of 292F g^−1^, which is attributed to particle agglomeration that partially occludes the microporous network and restricts ion accessibility, as corroborated by TEM and BET analyses. However, the electrochemical performance cannot be attributed solely to Au content. Similarly, ACAu-20–20-ul achieves a moderate capacitance enhancement (320F g^−1^), implying that insufficient irradiation duration limits complete Au deposition, resulting in suboptimal surface coverage and incomplete formation of conductive pathways. It is suggested that an appropriate balance among Au content, nanoparticle size and dispersion, structural disorder, and pore accessibility is important for achieving enhanced capacitive performance. Acoustic cavitation generates intense local micro-mixing and enhances mass transfer in the liquid phase, which can facilitate the transport of Au precursor species toward the carbon surface [50]. These effects may provide a more homogeneous environment for nanoparticle nucleation and limit subsequent particle growth and coalescence [51], consistent with the smaller and more uniformly distributed AuNPs observed under ultrasonic irradiation.

**Fig. 6:**
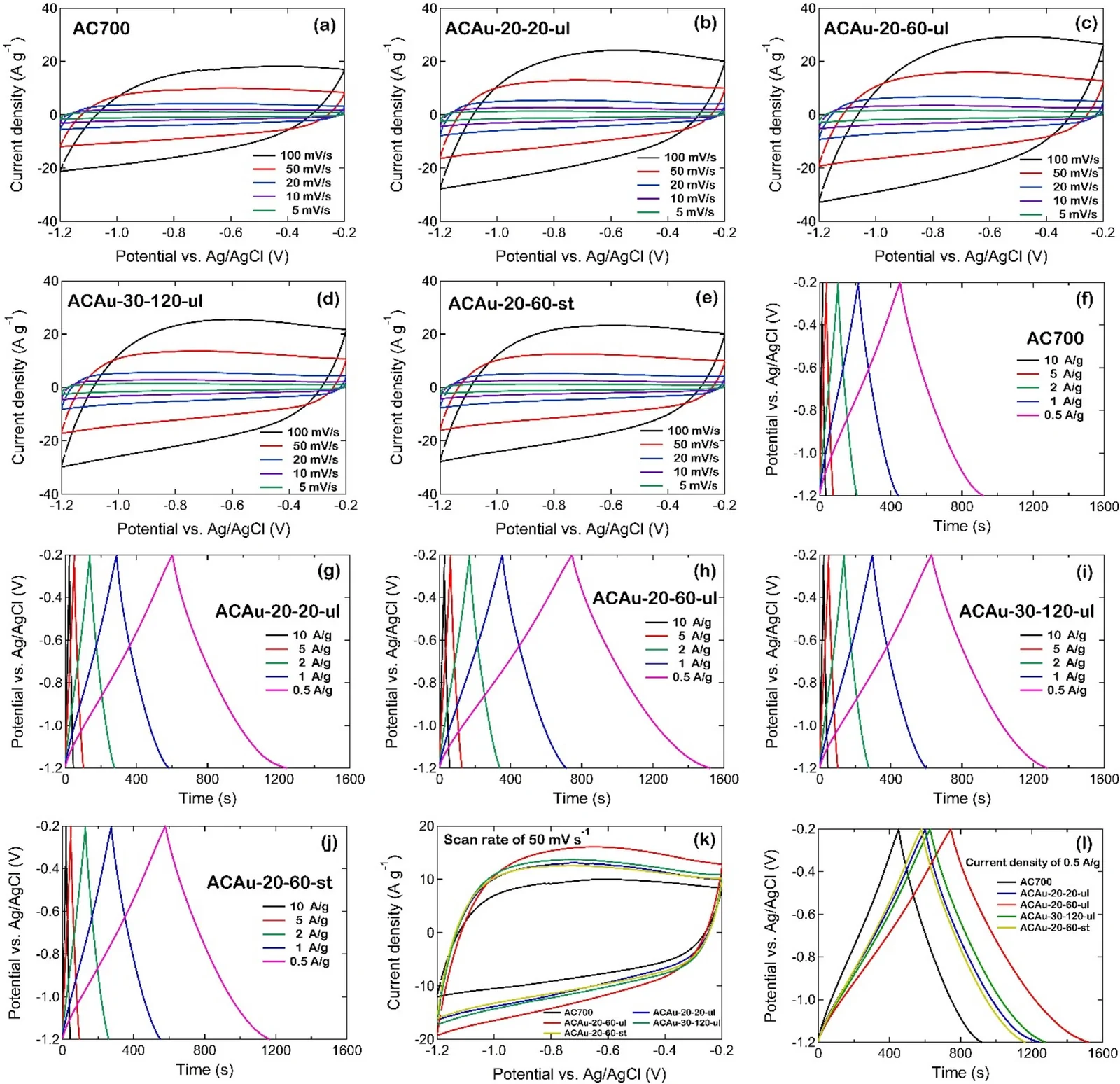
CV curves at various scan rates (5 – 100 mV s^−1^) of (a) AC700, (b) ACAu-20–20-ul, (c) ACAu-20–60-ul, (d) ACAu-30–120-ul, and (e) ACAu-20–60-st; GCD curves at various current densities (0.5 – 10 A g^−1^) of (f) AC700, (g) ACAu-20–20-ul, (h) ACAu-20–60-ul, (i) ACAu-30–120-ul, and (j) ACAu-20–60-st; (k) comparative CV curves at 50 mV s^−1^ and (l) comparative GCD curves at 0.5 A g^−1^ of all samples.

**Table 2:** Electrochemical performance comparison of Au-decorated carbon-based supercapacitor electrode materials.

Materials	Electrolyte	Current density (A g^−1^)	Specific capacitance in a 3-electrode system (F g^−1^)	Specific capacitance in a 2-electrode system (F g^−1^)	Energy density / Power density (Wh kg^−1^ / W kg^−1^)	Ref.
GQDs / Au	3 M KOH	1 A g^−1^	480	−	10.6 / 106	[29]
Au-PPCM	6 M KOH	0.5 A g^−1^	440	140.3	19.5 / 499.9	[31]
PC@AuNP-1	6 M KOH	0.5 A g^−1^	421.4	75	10.4 / 124.9	[54]
AC700	6 M KOH	0.5 A g^−1^	233	100	13.9 / 490	This work
ACAu-20–60-ul	6 M KOH	0.5 A g^−1^	387	160	22.2 / 500	This work
ACAu-20–60-st	6 M KOH	0.5 A g^−1^	292	121	16.8 / 500	This work

The rate performance and extended cycling durability of the fabricated electrodes were subsequently examined, with the corresponding results presented in Fig. 7. The variation of specific capacitance as a function of applied current density for all samples is depicted in Fig. 7a. Consistent with the expected behavior of microporous carbon-based electrode materials, the specific capacitance of all samples undergoes a gradual decline with increasing current density over the range of 0.5 to 50 A g^−1^, a phenomenon widely ascribed to the kinetic constraints imposed on ion diffusion at elevated current densities [52]. Throughout the entire current density range investigated, ACAu-20–60-ul consistently achieves the highest specific capacitance values, delivering 387F g^−1^ at 0.5 A g^−1^ and maintaining approximately 210F g^−1^ at 50 A g^−1^, thereby demonstrating outstanding rate capability. In contrast, AC700 exhibits the lowest capacitance throughout, retaining only ∼ 90F g^−1^ at 50 A g^−1^, reflecting its limited electrical conductivity in the absence of conductive modifiers. The long-term cycling stability and coulombic efficiency of selected electrodes were evaluated over 5000 consecutive charge–discharge cycles at a current density of 10 A g^−1^, as presented in Fig. 7b–7e. Pristine AC700 (Fig. 7b) retains 92.1% of its initial capacitance after 5000 cycles with a coulombic efficiency of 95.0%, indicating acceptable structural stability but relatively limited charge–discharge reversibility. Upon Au incorporation, all samples exhibit improved cycling stability and coulombic efficiency, confirming that the introduction of AuNPs enhances both the structural robustness and reversibility of the electrodes. Among all samples, ACAu-20–60-ul (Fig. 7c) achieves the highest capacitance retention of 96.5% after 5000 cycles, accompanied by an outstanding coulombic efficiency of 98.9%, which represents a marked improvement over pristine AC700 (95.0%). This superior cycling performance is ascribed to the synergistic effect of uniformly dispersed AuNPs and the well-preserved microporous carbon framework. The homogeneous Au distribution not only enhances electrical conductivity but also reinforces the structural integrity of the electrode, effectively suppressing performance degradation during prolonged charge–discharge cycling. ACAu-30–120-ul (Fig. 7d) exhibits a capacitance retention of 93.8% with a coulombic efficiency of 96.3%, which is lower than that of ACAu-20–60-ul. ACAu-20–60-st (Fig. 7e) displays a coulombic efficiency of 97.5% but a relatively lower capacitance retention of 92.4% after 5000 cycles, comparable to that of pristine AC700. The aggregation of AuNPs in the magnetic stirring-prepared sample likely generates localized mechanical stress and induces partial pore blockage, which accelerates performance degradation over prolonged operation. Collectively, these findings confirm that both rate capability and long-term cycling stability are critically governed by the dispersion quality of AuNPs and the structural integrity of the carbon framework.

**Fig. 7:**
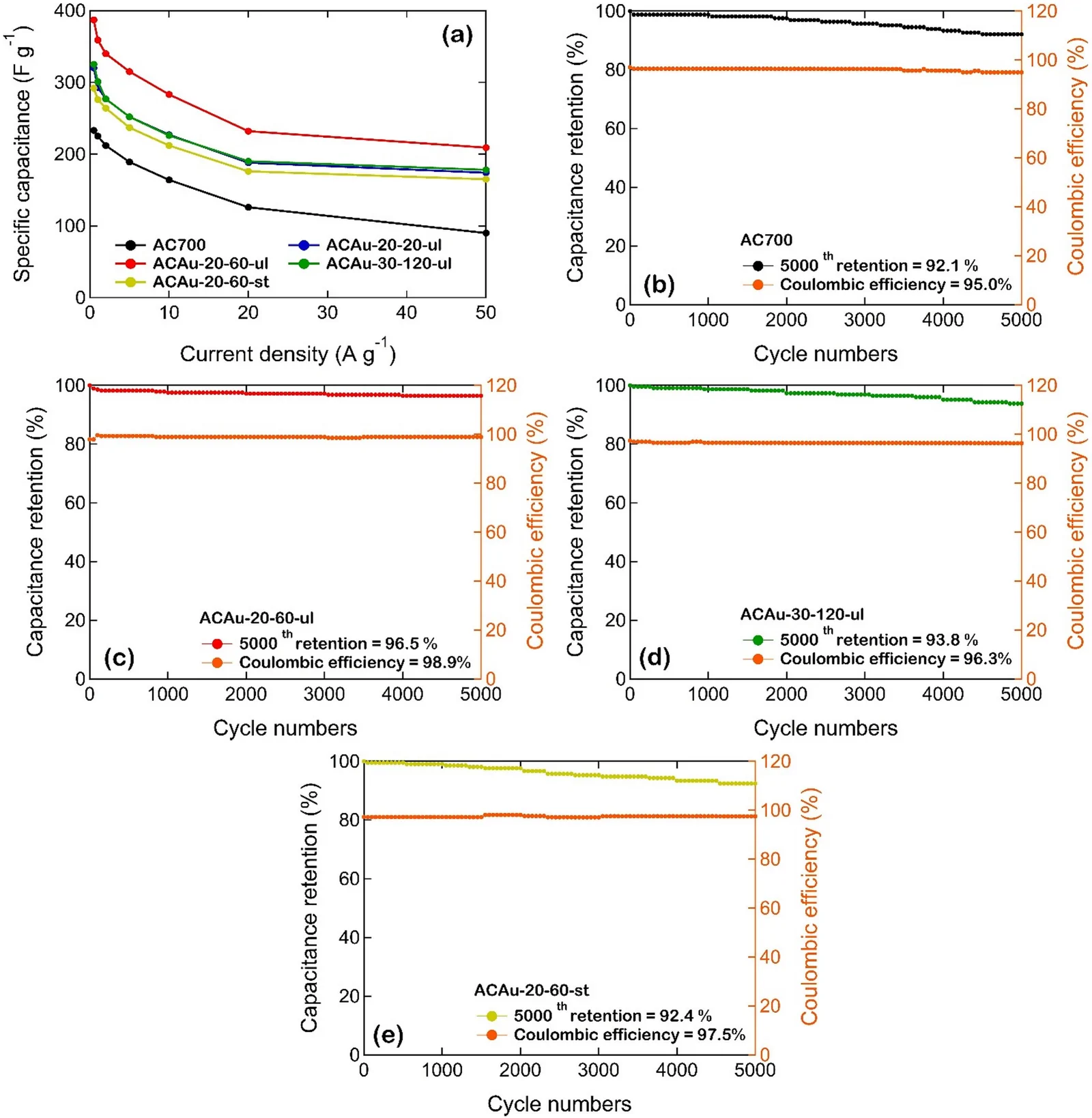
(a) Rate capability curves of all samples; capacitance retention and coulombic efficiency over 5000 charge–discharge cycles at 10 A g^−1^ for (b) AC700, (c) ACAu-20–60-ul, (d) ACAu-30–120-ul, and (e) ACAu-20–60-st.

### 3.4 Electrochemical properties of Au-decorated activated carbon in a two-electrode configuration

The electrochemical behavior of selected symmetric supercapacitor devices was examined in a two-electrode cell arrangement employing 6 M KOH as the electrolyte, with the obtained results illustrated in Fig. 8. The CV profiles of all assembled devices (Fig. 8a–8c) were acquired over an operational voltage range of − 0.5 to 0.5 V at scan rates spanning from 5 to 100 mV s^−1^. At relatively low scan rates (5–20 mV s^−1^), all devices exhibit nearly rectangular CV shapes, indicative of predominantly EDLC-type charge storage behavior. With increasing scan rate, however, a gradual deviation from the ideal rectangular geometry becomes apparent, a characteristic feature associated with ion diffusion constraints within the microporous electrode architecture under high sweep rate conditions. Of particular note, ACAu-20–60-ul (Fig. 8b) maintains a well-preserved CV profile over the entire range of scan rates examined, reflecting enhanced charge transport kinetics and reduced internal resistance relative to both AC700 (Fig. 8a) and ACAu-20–60-st (Fig. 8c). A side-by-side comparison of the CV profiles acquired at 50 mV s^−1^ (Fig. 8g) unambiguously highlights the electrochemical performance disparities among the assembled devices, with ACAu-20–60-ul displaying the largest enclosed CV area, corroborating its superior capacitive response in the two-electrode device configuration. ACAu-20–60-st displays an intermediate CV area, while AC700 exhibits the smallest, reflecting the progressive benefit of AuNPs incorporation on charge storage capability. It is also worth noting that all three samples display similar CV curve shapes without prominent redox peaks, further confirming the dominance of double-layer charge storage in the symmetric device configuration. The GCD profiles of individual devices (Fig. 8d–8f) display nearly symmetric triangular shapes across all current densities from 0.5 to 10 A g^−1^, confirming good electrochemical reversibility and high coulombic efficiency. A discernible voltage drop (IR drop) at the onset of the discharge branch is observed for all samples, which is most pronounced in AC700 (Fig. 8d), reflecting its relatively higher internal resistance in the absence of conductive AuNPs. The discharge time increases markedly upon Au incorporation, with ACAu-20–60-ul (Fig. 8e) exhibiting the longest discharge time at all applied current densities. The discharge curve of ACAu-20–60-st (Fig. 8f) shows a nonlinear curvature during discharge at 0.5 A g^−1^, suggesting a higher degree of ion transport resistance compared to ACAu-20–60-ul, consistent with the particle aggregation behavior observed by TEM analysis. The comparative GCD curves at 0.5 A g^−1^ (Fig. 8h) reveal that ACAu-20–60-ul possesses the longest discharge time (∼160 s), followed by ACAu-20–60-st (∼120 s) and AC700 (∼100 s). The specific capacitance values of the symmetric devices at various current densities are compiled in Table 2. At 0.5 A g^−1^, ACAu-20–60-ul achieves the highest device-level specific capacitance of 160F g^−1^, while ACAu-20–60-st and AC700 deliver 121 and 100F g^−1^, respectively, corresponding to enhancements of 60% and 21% over AC700 for the two Au-decorated samples. The rate capability curves (Fig. 8i) confirm that all samples undergo progressive capacitance decay with increasing current density, as expected for microporous carbon-based electrodes operating under kinetic limitations at high rates. ACAu-20–60-ul demonstrates the most favorable rate performance, retaining approximately 90F g^−1^ (∼56% retention) at 50 A g^−1^ relative to its value at 0.5 A g^−1^. In contrast, AC700 retains only ∼ 20F g^−1^ (∼20% retention) at 50 A g^−1^, reflecting its significantly higher rate-dependent performance degradation. ACAu-20–60-st exhibits intermediate rate behavior, retaining approximately 30F g^−1^ (∼25% retention) at 50 A g^−1^. These results confirm that ACAu-20–60-ul represents the optimal electrode composition for practical symmetric supercapacitor device applications, and that ultrasound-assisted deposition at moderate conditions is a superior strategy compared to conventional stirring for fabricating high-performance Au-decorated carbon electrodes.

**Fig. 8:**
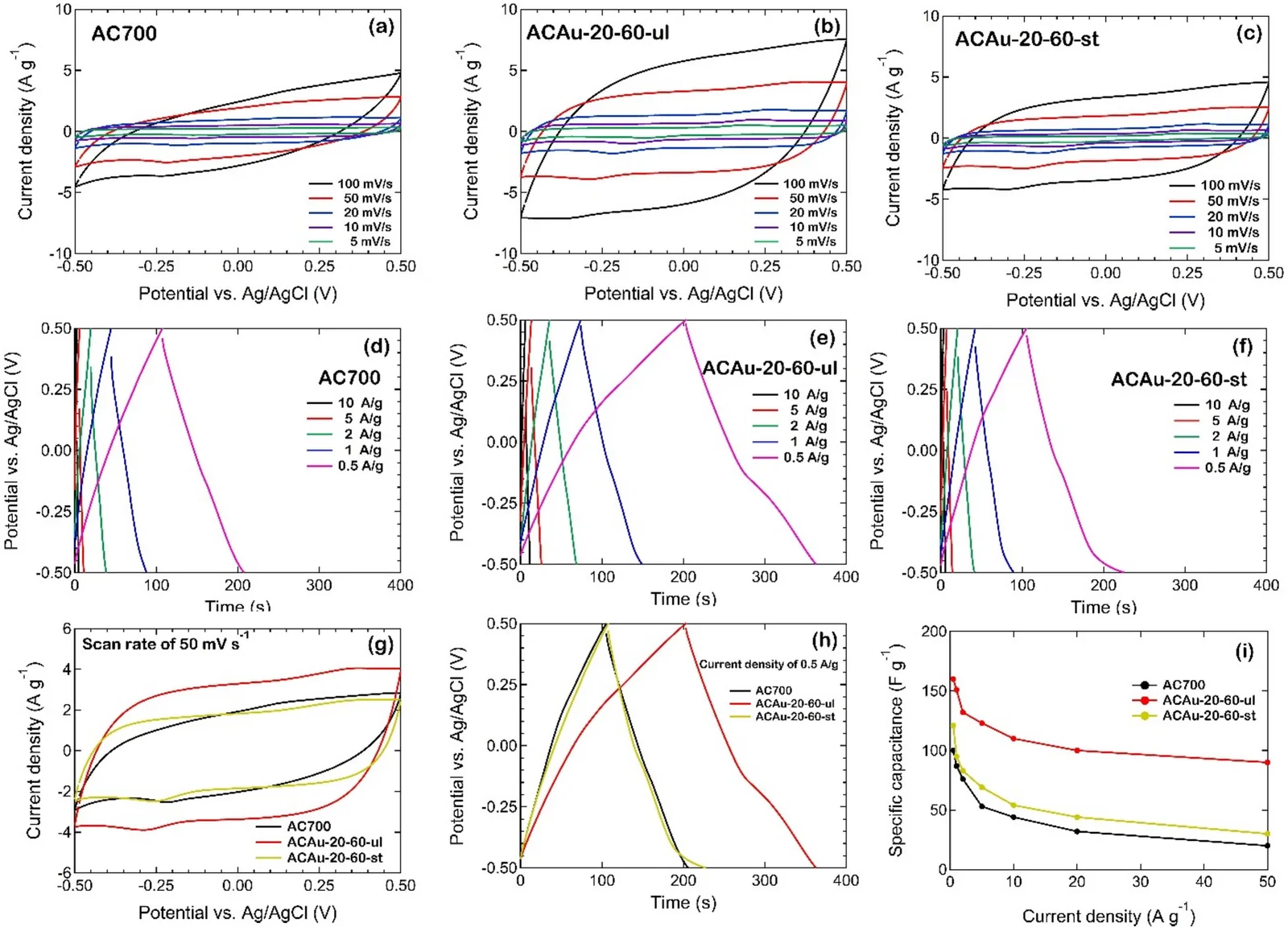
Electrochemical performance of symmetric supercapacitor devices in two-electrode configuration: CV curves at various scan rates (5 – 100 mV s^−1^) of (a) AC700, (b) ACAu-20–60-ul, and (c) ACAu-20–60-st; GCD curves at various current densities (0.5 – 10 A g^−1^) of (d) AC700, (e) ACAu-20–60-ul, and (f) ACAu-20–60-st; (g) comparative CV curves at 50 mV s^−1^, (h) comparative GCD curves at 0.5 A g^−1^, and (i) rate capability curves of all samples.

To gain deeper insight into the charge storage mechanism and ion transport behavior of the fabricated symmetric supercapacitor devices, electrochemical impedance spectroscopy (EIS) analysis was performed, and the resulting Nyquist plots are presented in Fig. 9a. The acquired impedance data were modeled using the equivalent circuit depicted in Fig. 9b, which consists of a solution resistance (R_s_) connected in series with a parallel arrangement of a capacitance (C_1_) and a charge transfer resistance (R_ct_), the latter being further coupled in series with a Warburg impedance (Z_w_), followed by a constant phase element (Q_1_) accounting for the capacitive response at low frequencies. All three samples exhibit a well-defined semicircular arc in the high-to-medium frequency domain, attributable to the charge transfer process occurring at the electrode–electrolyte interface, succeeded by a near-vertical tail in the low-frequency regime, which is characteristic of ideal capacitive behavior [53]. The solution resistance (R_s_), which encapsulates the collective contributions of the electrolyte resistance, intrinsic electrode resistance, and contact interface resistance, remains largely consistent across all samples, recording values of 1.64, 1.68, and 1.79 Ω for AC700, ACAu-20–60-ul, and ACAu-20–60-st, respectively, implying that Au incorporation exerts negligible influence on the bulk electrolyte resistance or interfacial contact properties of the devices. The charge-transfer resistance (R_ct_), inferred from the diameter of the semicircular arc in the high-frequency region, varies considerably among the samples. AC700 exhibits the highest R_ct_ of 4.7 Ω, whereas Au decoration reduces the R_ct_ to 0.8 Ω for ACAu-20–60-ul and 0.9 Ω for ACAu-20–60-st, corresponding to reductions of approximately 83% and 81%, respectively. A comparable reduction in R_ct_ (∼85%) has also been reported for Au-decorated porous carbon [31], supporting the favorable effect of Au incorporation on interfacial charge-transfer behavior. The substantially lower R_ct_ values of the Au-decorated samples therefore indicate more favorable interfacial charge-transfer kinetics following Au incorporation. The energy density and power density characteristics of the assembled symmetric supercapacitor devices were extracted from GCD measurements and are graphically represented in the Ragone plot (Fig. 9c). ACAu-20–60-ul achieves the highest energy density of 22.2 Wh kg^−1^ at a power density of 500 W kg^−1^, while still retaining 12.5 Wh kg^−1^ at an exceptionally high-power density of 50,000 W kg^−1^, underscoring its remarkable capability to sustain both high energy and high-power output simultaneously. By comparison, AC700 attains a peak energy density of merely 13.9 Wh kg^−1^ at 499 W kg^−1^, which drops sharply to 2.8 Wh kg^−1^ at 50,400 W kg^−1^, highlighting the severe rate-dependent energy dissipation associated with the unmodified carbon electrode. ACAu-20–60-st demonstrates intermediate energy storage behavior, yielding a maximum energy density of 16.8 Wh kg^−1^ at 500 W kg^−1^ and preserving 4.2 Wh kg^−1^ at 50,400 W kg^−1^, a performance level that surpasses that of AC700 yet remains inferior to ACAu-20–60-ul over the full power density range evaluated. The energy and power density values of ACAu-20–60-ul were further benchmarked against previously reported Au-decorated carbon materials in the literature. As shown in Fig. 9c, ACAu-20–60-ul demonstrates competitive performance relative to GQDs/Au (10.6 Wh kg^−1^ at 106 W kg^−1^ in 3 M KOH) [29], Au-PPCM (19.5 Wh kg^−1^ at 499.9 W kg^−1^ in 6 M KOH) [31], and PC@AuNP-1 (10.4 Wh kg^−1^ at 124.9 W kg^−1^ in 6 M KOH) [54]. Notably, ACAu-20–60-ul achieves a higher maximum energy density than GQDs/Au and PC@AuNP-1, and maintains superior energy retention at high power densities compared to all referenced materials, highlighting the advantage of the ultrasound-assisted deposition approach in optimizing both energy storage capacity and rate capability. Although Au-PPCM exhibits a slightly higher maximum energy density, ACAu-20–60-ul is prepared from a low-cost biomass-derived precursor (rice straw) and employs a straightforward synthesis route, rendering it a more practical and sustainable alternative for high-performance supercapacitor applications.

**Fig. 9:**
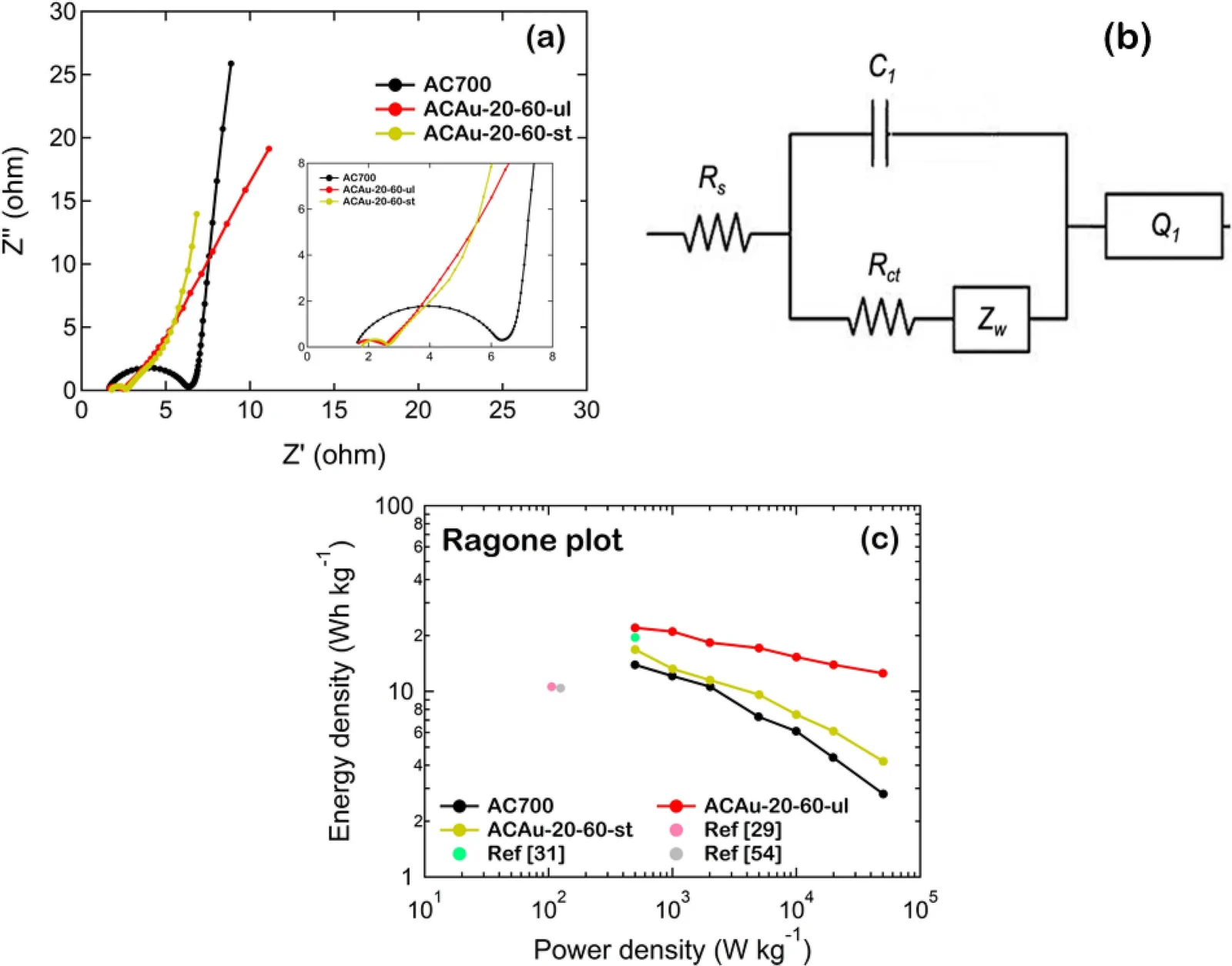
(a) Nyquist plots of AC700, ACAu-20–60-ul, and ACAu-20–60-st, (b) equivalent circuit model used for EIS data fitting; (c) Ragone plot comparing the energy and power densities of the assembled symmetric supercapacitor devices with previously reported Au-decorated carbon materials.

## 4 Conclusions

AuNPs were successfully deposited onto rice-straw-derived activated carbon using ultrasound-assisted irradiation and conventional magnetic stirring. The ultrasound-only control experiment demonstrated that prolonged ultrasonic irradiation increased structural disorder without improving electrochemical performance. Instead, the principal contribution of ultrasound was associated with acoustic cavitation during AuNP deposition. Cavitation-induced micro-mixing and enhanced interfacial mass transfer promoted Au precursor transport and nucleation while suppressing excessive particle growth and coalescence. Consequently, ultrasound-assisted deposition produced smaller and more uniformly distributed AuNPs, with a mean particle size of 26.88 nm compared with 81.79 nm for conventional magnetic stirring. XRD and XPS confirmed the formation of crystalline metallic Au⁰ nanoparticles, while the high surface area and microporous structure of the activated carbon were well preserved. The optimized ACAu-20–60-ul electrode delivered a specific capacitance of 387F g^−1^ at 0.5 A g^−1^, representing a 66% enhancement over pristine AC700. In the symmetric two-electrode device, ACAu-20–60-ul achieved 160F g^−1^ at 0.5 A g^−1^ and a maximum energy density of 22.2 Wh kg^−1^ at 500 W kg^−1^, together with 96.5% capacitance retention after 5000 cycles, a coulombic efficiency of 98.9%, and a low charge-transfer resistance of 0.8 Ω. These findings demonstrate that the beneficial role of ultrasound primarily arises from cavitation-mediated control of AuNP formation and dispersion rather than direct modification of the carbon substrate. Therefore, ultrasound-assisted deposition provides an effective sonochemical strategy for controlling metal–carbon interfaces and enhancing the electrochemical performance of biomass-derived porous carbon electrodes.

## CRediT authorship contribution statement

**Tippapon Tocuweang:** Writing – original draft, Methodology, Investigation. **Darunee Aussawasathien:** Methodology, Conceptualization. **Hirokazu Okawa:** Methodology, Conceptualization. **Nakorn Worasuwannarak:** Writing – review & editing, Supervision, Conceptualization.

## Declaration of competing interest

The authors declare that they have no known competing financial interests or personal relationships that could have appeared to influence the work reported in this paper.
